# Structures of Chlorophyll Catabolites in Bananas (*Musa acuminata)* Reveal a Split Path of Chlorophyll Breakdown in a Ripening Fruit

**DOI:** 10.1002/chem.201201023

**Published:** 2012-07-16

**Authors:** Simone Moser, Thomas Müller, Andreas Holzinger, Cornelius Lütz, Bernhard Kräutler

**Affiliations:** a Institute of Organic Chemistry, University of Innsbruck6020 Innsbruck (Austria); b Center for Molecular Biosciences (CMBI), University of Innsbruck6020 Innsbruck (Austria); c Institute of Botany, University of Innsbruck6020 Innsbruck (Austria); d Present address: Institute of Chemical Sciences and Engineering, Ecole Polytechnique Fédérale de Lausanne1015 Lausanne (Switzerland)

**Keywords:** catabolite, chlorophyll, fruit, luminescence, pigment, structure elucidation

## Abstract

**Abstract:**

The disappearance of chlorophyll is a visual sign of fruit ripening. Yet, chlorophyll breakdown in fruit has hardly been explored; its non-green degradation products are largely unknown. Here we report the analysis and structure elucidation of colorless tetrapyrrolic chlorophyll breakdown products in commercially available, ripening bananas (*Musa acuminata*, Cavendish cultivar). In banana peels, chlorophyll catabolites were found in an unprecedented structural richness: a variety of new fluorescent chlorophyll catabolites (FCCs) and nonfluorescent chlorophyll catabolites (NCCs) were detected. As a rule, FCCs exist only "fleetingly" and are hard to observe. However, in bananas several of the FCCs (named *Mc*-FCCs) were persistent and carried an ester function at the propionate side-chain. NCCs were less abundant, and exhibited a free propionic acid group, but functional modifications elsewhere. The modifications of NCCs in banana peels were similar to those found in NCCs from senescent leaves. They are presumed to be introduced by enzymatic transformations at the stage of the mostly unobserved, direct FCC-precursors. The observed divergent functional group characteristics of the *Mc*-FCCs versus those of the *Mc*-NCCs indicated two major "late" processing lines of chlorophyll breakdown in ripening bananas. The "last common precursor" at the branching point to either the persistent FCCs, or towards the NCCs, was identified as a temporarily abundant "secondary" FCC. The existence of two "downstream" branches of chlorophyll breakdown in banana peels, and the striking accumulation of persistent *Mc*-FCCs call for attention as to the still-elusive biological roles of the resulting colorless linear tetrapyrroles.

## Introduction

Breakdown of chlorophyll is a major contributor to the diagnostic color changes in fall leaves.[1] De-greening and the typical changes of the colors of leaves are considered, first of all, as visible symptoms of senescence,[2,3] a form of programmed cell-death in plants.[4] Many fruit also de-green during ripening and degradation of chlorophyll is an easily observed sign of ripening.[5]

In spite of broad research efforts, the "disappearance" of chlorophyll was a biological puzzle for a long time,[6] since its products remained concealed.[7] Indeed, a breakthrough in this area was provided by the identification and structure elucidation of a colorless tetrapyrrole in senescent leaves of barley (*Hordeum vulgare*), named *Hv*-NCC-1.[1,8] Meanwhile, identification of the structures of relevant chlorophyll catabolites,[9,10] of key enzymes and their genes,[11–13] has resulted in general insights into the basic processes involved in chlorophyll breakdown in higher plants.[13,14] Colorless "nonfluorescent chlorophyll catabolites" (NCCs) are typically found (to accumulate) in senescent leaves and have been suggested to represent (with notable exceptions[15–19]) the "final stage" of chlorophyll breakdown (see Figure 1).[13] Likewise, ripening apples and pears contained known NCCs, suggesting a "common path" of chlorophyll breakdown in leaf senescence and fruit ripening.[5,20] Surprisingly, in (the peels of) ripening bananas (*Musa acuminata*, Cavendish cultivar) chlorophylls fade to give fluorescent chlorophyll catabolites (FCCs) mainly, and yellow bananas glow blue, when analyzed under UV light.[21,22]

**1:**
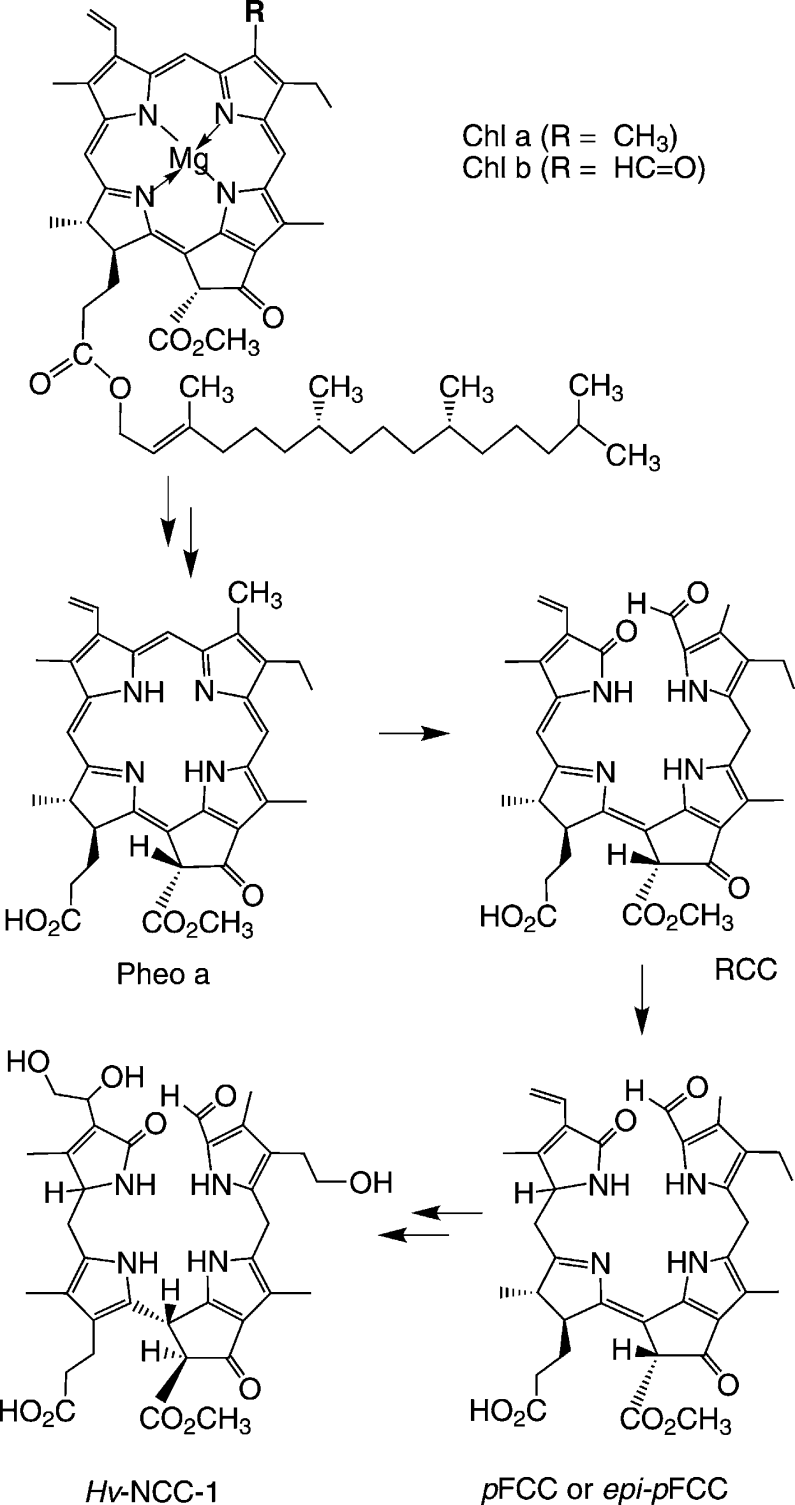
Structural outline of the "common" main path of chlorophyll breakdown in senescent leaves and ripening fruit.[13] Chlorophylls are degraded in the chloroplast by enzyme catalyzed processes via pheophorbide a (Pheo a) and the red chlorophyll catabolite (RCC) to "primary" fluorescent chlorophyll catabolites (*p*FCC, or its C1 epimer, *epi*-*p*FCC). FCCs with a free propionic acid group isomerize in the vacuole by an acid-catalyzed reaction to the corresponding nonfluorescent chlorophyll catabolites (NCCs). *Hv*-NCC-1 is the main NCC in senescent leaves of barley (*Hordeum vulgare*) and derived from *p*FCC.[8]

The "disappearance" of chlorophyll in the peels of ripening bananas has attracted considerable interest, as expected in view of the important commercial role of bananas. In one such study, two independent degradation pathways of the enzymatic breakdown of chlorophyll in Cavendish bananas were suggested.[23] One of them was proposed to proceed through the so-called "Type I" degradation path leading to modified-green chlorophylls, whereas non-green catabolites were not identified in that study.[23] Other recent reports dealt with the gene expression in the peels of ripening bananas. So far, enzymes assigned to proper de-greening reactions have remained elusive.[24–27] However, in one of these studies, an NCC was tentatively identified by a UV spectrum, and was named M-NCC-1.[24]

A closer look at the degradation of chlorophyll in ripening bananas revealed a stunning variety of colorless chlorophyll catabolites, as the result of a surprisingly complex breakdown path. Among the catabolites were four persistent FCCs (*Mc*-FCCs), which accumulated in the peels of ripe bananas.[21,22] They had unprecedented complex structures and were typified as "hyper-modified" FCCs (*hm*FCCs). Part of chlorophyll catabolism in bananas was thus indicated to deviate from the suggested "common" path of chlorophyll breakdown, which furnishes nonfluorescent chlorophyll catabolites (NCCs) as its typical "final" tetrapyrrolic products.[13] However, the structures of many of the presumed chlorophyll catabolites in bananas remains yet to be elucidated. We have identified most of these catabolites, as well as their structures and their changing occurrence during the ripening process, as reported here.

## Results

**Occurrence of chlorophyll catabolites in the peels of ripening bananas**: Freshly prepared extracts of the peels of commercial ethylene-ripened bananas (*Musa acuminata*, Cavendish cultivar, short: *Mc*-bananas) were analyzed for their chlorophyll catabolites by high performance liquid chromatography (HPLC), with UV absorbance and fluorescence detection. At the early ripening stage, (rs) 5 (see, for example, ref. [28) about a dozen *Mc*-FCCs were tentatively identified directly on the basis of their absorbance and fluorescence characteristics, in addition to ten *Mc*-NCCs (see Figure 2). Three minor yellow fractions were also found, and typified as yellow chlorophyll catabolites (YCCs).[17] To describe the major colorless chlorophyll catabolites from the banana peels, they were first classified as FCCs or as NCCs, and specified further as *Mc*-FCC-*t*_R_ or *Mc*-NCC-*t*_R_ (*t*_R_ is their retention time in minutes under the conditions of a standardized analytical HPLC experiment (see ref. [21,22).

**2:**
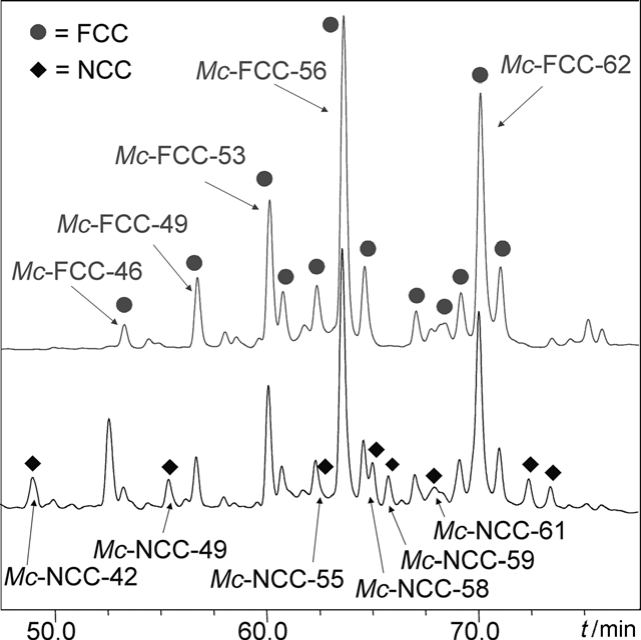
HPLC analysis of chlorophyll catabolites in an extract of peels of freshly ripened bananas. Commercially available *Musa acuminata* (Cavendish cultivar, Chiquita brand) at ripening stage (rs) 5 were analyzed with online detection by absorbance at 320 nm (lower trace) and emission at 450 nm (upper trace); dots and diamonds indicate fractions classified as FCCs and NCCs, respectively, and which are indexed according to the retention times observed under conditions of a standard analytical HPLC experiment (solvent composition A1–for further details see the Supporting Information, Figure S1).

The abundance of these chlorophyll catabolites varied according to the extent of the ripening of the bananas, as described below. The distribution of the catabolites also varied locally, in particular near "senescence-associated" dark spots appearing at the stages of over-ripening.[22] From extracts of two batches of peels of freshly ripe bananas (65 and 99 bananas of Chiquita or Dole brands, weighing about 13 and 20 kg, respectively), the most abundant six FCC and seven NCCs fractions were collected and used for further spectroscopic characterization (see Figure 2, and Tables 1 and 2). The main FCC- and NCC-fractions were separated by two (sometimes by three) consecutive runs of preparative HPLC (see the Experimental Section), and their structures were analyzed, as described below. 
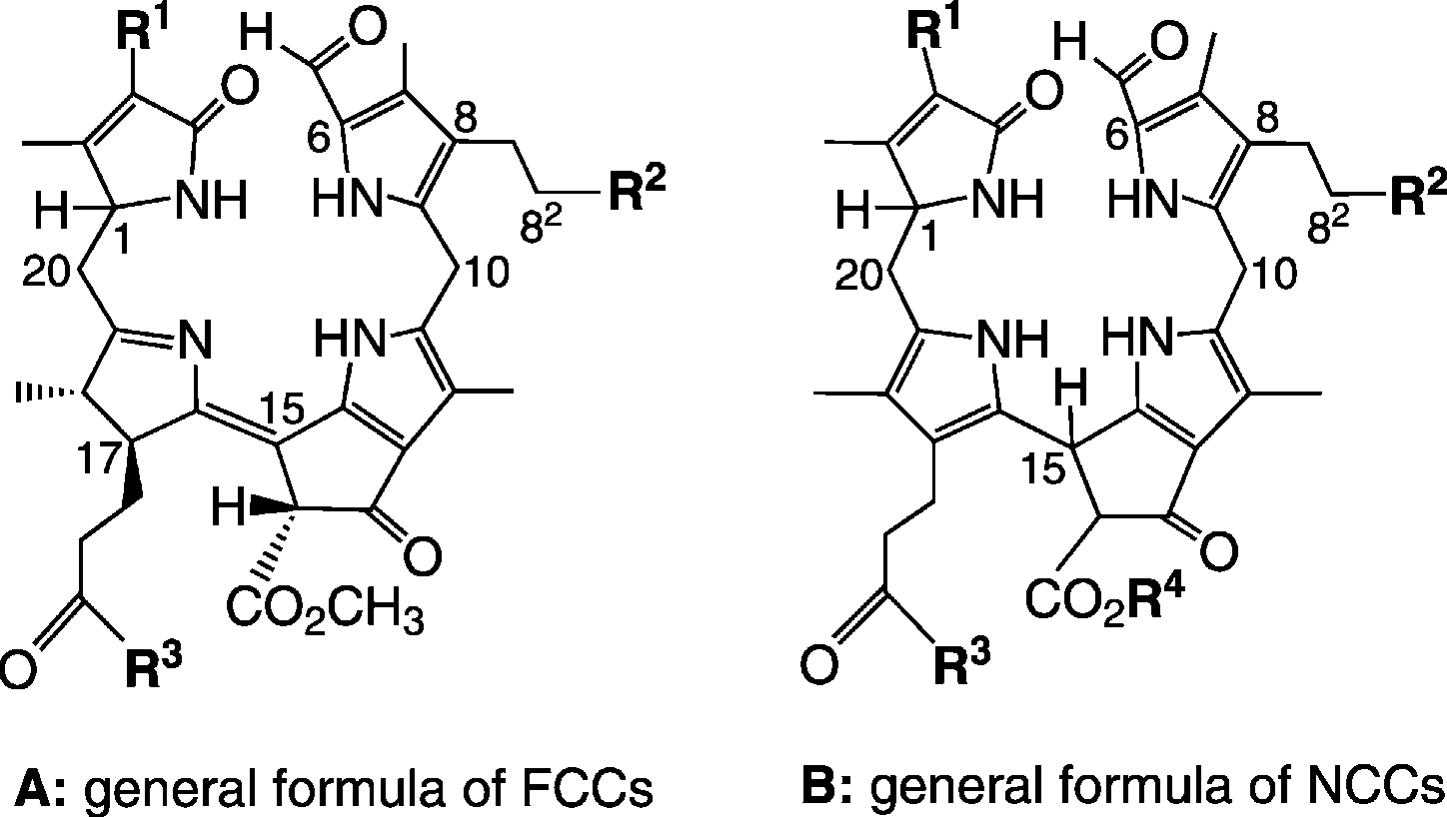


**1:** List of fluorescent chlorophyll catabolites (*Mc*-FCCs) observed in extracts from peels of ripening bananas (*Musa acuminata*). The common general formula A is depicted below with specification of the observed covalent modifications at positions R^1^, R^2^, and R^3^.

Compound	R^1^	R^2^	R^3^
*Mc*-FCC-46	CH=CH_2_	*O*-Glc^[a]^	daucic acid^[b]^
*Mc*-FCC-49	CH=CH_2_	*O*-Glc^[a]^	daucic acid^[c]^
*Mc*-FCC-53	CH=CH_2_	OH	daucic acid^[b]^
*Mc*-FCC-56	CH=CH_2_	OH	daucic acid^[c]^
*Mc*-FCC-62	CH=CH_2_	OH	OH
*Mc*-FCC-71^[d]^	CH=CH_2_	OH	OCH_3_

[a] Glc=β-glucopyranosyl, [b] bound at 5′-OH [c] bound at 4′-OH; [d] likely to be an artifact, from methanolysis of the "persistent" FCC-daucyl-esters *Mc*-FCC-56 and *Mc*-FCC-53; see the main text for details.

**2:** List of nonfluorescent chlorophyll catabolites (*Mc*-NCCs) observed in extracts of peels of ripening bananas (*Musa acuminata*). The common general formula B is depicted above with specification of observed covalent modifications at positions R^1^, R^2^, R^3^, and R^4^.

Compound	R^1^	R^2^	R^3^	R^4^
*Mc*-NCC-26	CH(OH)-CH_2_OH	OH	OH	H
*Mc*-NCC-42^[a]^	CH(OH)-CH_2_OH	OH	OH	CH_3_
*Mc*-NCC-49	CH=CH_2_	OH	OH	H
*Mc*-NCC-59	CH=CH_2_	*O*-Glc^[b]^	OH	CH_3_
*Mc*-NCC-61	CH=CH_2_	OH	OH	CH_3_
*Mc*-NCC-55	CH=CH_2_	OH	daucic acid^[c]^	CH_3_
*Mc*-NCC-58	CH=CH_2_	OH	daucic acid^[c]^	CH_3_

[a] Identified with *So*-NCC-2 from senescent spinach leaves^[1]^; [b] Glc=β-glucopyranosyl; [c] Daucic acid bound at 5′-OH or at 4′-OH.

**Fluorescent catabolites (*Mc*-FCCs) in extracts of the peels of freshly ripe bananas**: From our previous work on chlorophyll catabolites from the peels of ripe bananas, the surprising accumulation of four polar "hyper-modified" FCCs (*hm*FCCs) is known. These *hm*FCCs were provisionally named *Mc*-FCC-46, *Mc*-FCC-49, *Mc*-FCC-53 and *Mc*-FCC-56, and their structures were established in detailed spectroscopic studies (see Figure 3).[21,22] A less polar FCC, *Mc*-FCC-62, was a significant further FCC-fraction in the peels of freshly bought bananas, which were still incompletely ripe (i.e., at rs=5; see Figure 2). From the peels of 65 bananas ("Dole" brand), a pure sample of about 115 μg of *Mc*-FCC-62 was obtained after three rounds of purification by preparative HPLC (see the Experimental Section). Mass spectrometric analysis of *Mc*-FCC-62 revealed its molecular formula as C_35_H_40_N_4_O_8_. *Mc*-FCC-62 was thus indicated to contain only one oxygen atom more than a "primary" FCC (*p*FCC)[29,30] and to be less complex than the more polar "hyper-modified" *Mc*-FCCs (e.g., *Mc*-FCC-56).[21,22] The structure of *Mc*-FCC-62 was elucidated with the help of homo- and heteronuclear NMR spectroscopic investigations, which indicated a hydroxyl group at the terminal position of the ethyl substituent at C8 (see the Experimental Section, Figure 7). *Mc*-FCC-62 was thus revealed to be a 8^2^-hydroxy-13^2^-(methoxycarbonyl)-3^1^,3^2^-didehydro-1,4,5,10,17,18,20,22-octahydro-4,5-seco-(22 H)-phytoporphyrin (see Table 1] and Figure 3).

**3:**
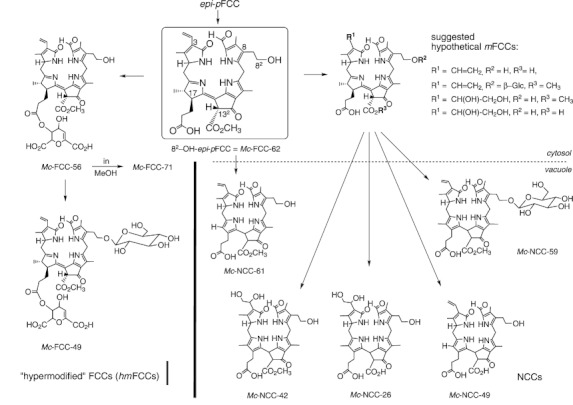
Suggested "later" steps of chlorophyll breakdown in the peels of ripening *Mc*-bananas, beginning with "*epi*-*p*FCC". *Mc*-FCC-62 (8^2^-hydroxy-*epi*-*p*FCC), the "secondary" FCC (*s*FCC) in *Mc*-bananas, accumulates early during ripening (at rs=5) and is proposed to be the common precursor of all "downstream" catabolites. "Hyper-modified" FCCs (*hm*FCCs) with a complex propionate ester function, such as *Mc*-FCC-56 and *Mc*-FCC-49, accumulate and represent one branch of chlorophyll breakdown. "Modified" FCCs (*m*FCCs) with a free propionic acid group are elusive and may only exist transiently: they are imported into the vacuole, where they isomerize rapidly to the corresponding NCCs by an acid-catalyzed reaction.[34] *Mc*-NCCs are the main observed products of the second branch of chlorophyll breakdown, and carry a free propionic acid function (see the main text for details).

The least polar FCC named *Mc*-FCC-71, which was detected in small amounts in fresh peel extracts (57 μg from 99 bananas), had a molecular formula of C_36_H_42_N_4_O_8_. It was characterized as 8^2^-hydroxy-13^2^-(methoxycarbonyl)-17^4^-methyl-3^1^,3^2^-didehydro-1,4,5,10,17,18,20,22-octahydro-4,5-seco-(22 H)-phytoporphyrin (see Table 1] and Figure 3). *Mc*-FCC-71 was thus identified as the methyl ester of *Mc*-FCC-62. As the natural occurrence of a methyl ester of an FCC was unprecedented, we examined the likelihood of the formation of *Mc*-FCC-71 during work-up and spectral analysis. In fact, the same compound was formed (with partial deuteration) when *Mc*-FCC-56, the major *hm*FCC, was dissolved in the NMR solvent CD_3_OD and the sample was kept at room temperature for several days during spectral analysis. The NMR sample was analyzed and separated by HPLC: a less polar FCC formed, which had the characteristics of *Mc*-FCC-71. It exhibited, in particular, a ^1^H NMR spectrum similar to that of *Mc*-FCC-71 (from the original extract), except for the absence of the propionate methyl ester singlet at *δ*=3.58 ppm, as well as partial loss of the signals assigned to H_2_C-20 and HC-1 (see Tables 1] and 2] for atom numbering). ESI-MS of the newly formed *Mc*-FCC-71 indicated extensive deuterium labeling by the mass distribution of the pseudo-molecular ions, giving a best fit for 4:17:41 and 38 % of the [D_3_]/[D_4_]/[D_5_] and [D_6_]-isotopomers, respectively (see the Supporting Information). The spectra thus identified this FCC-sample as [D_*n*(*n*=3−6)_]-*Mc*-FCC-71, in which a trideutero-methyl ester was present. In addition, ^1^H atoms at C1 and C20 were also partially replaced by ^2^ H. *Mc*-FCC-71 was thus indicated to represent an isolation artifact and to be formed in the extract by a trans-esterification of *hm*FCCs (such as *Mc*-FCC-56) by MeOH.

**Nonfluorescent catabolites (*Mc*-NCCs) in extracts of the peels of ripe bananas**: In fresh extracts of the peels of ripening *Mc*-bananas, ten NCC fractions were detected by analytical HPLC (solvent composition A1, see the Supporting Information) and classified tentatively, based on their UV spectra. Seven of the *Mc*-NCCs were isolated by preparative HPLC, and further analyzed by mass spectrometry and NMR spectroscopy (see the Experimental Section).

*Mc*-NCC-26 was the most polar NCC identified in extracts from yellow banana peels. From mass spectrometric analysis, the molecular formula of *Mc*-NCC-26 was deduced as C_34_H_40_N_4_O_10_, providing the basis for a tentative structure assignment. *Mc*-NCC-26 was indicated to have no ester function at C(13^2^), but a 3^1^,3^2^-dihydroxyethyl group (at C3) and a 8^2^-hydroxyethyl group (at C8 of the tetrapyrrolic NCC core, see Table 2] and Figure 3).

A slightly less polar NCC, *Mc*-NCC-42, was a significant fraction in most extracts from peels of yellow bananas (0.89 μmol (600 μg) from 65 bananas; see Figure 2). Mass spectra indicated *Mc*-NCC-42 to have the molecular formula of C_35_H_42_N_4_O_10_. The molecular constitution of *Mc*-NCC-42 was elucidated with the help of homo- and hetero-nuclear NMR-correlations (see the Experimental Section, Figure 8). It was thus identified as a 3^1^,3^2^,8^2^-trihydroxy-13^2^-methoxycarbonyl-1,4,5,10,15,20,24-octahydro-4,5-seco-(22 H,24 H)-phytoporphyrin, and was revealed to carry 3^1^,3^2^-dihydroxyethyl and 8^2^-hydroxyethyl groups, respectively, as side chains at C-3 and C-8 of the NCCs tetrapyrrole core (see Table 2] and Figure 3). It thus had the same molecular constitution as *Hv*-NCC-1, the NCC originally isolated from senescent leaves of barley (*Hordeum vulgare*).[8,31] *Mc*-NCC-42 also had the same constitution as an NCC found in senescent leaves of spinach (*Spinacia oleracea*), called *So*-NCC-2, and described as the (C1)-epimer of *Hv*-NCC-1.[32] Indeed, the identity of *Mc*-NCC-42 with *So*-NCC-2 was deduced from a combination of its NMR data and by a HPL-chromatographic comparison (see below).

The next less polar NCC, *Mc*-NCC-49, a rather abundant fraction in most extracts, was isolated from peels of 99 yellow bananas (0.35 μmol (226 μg)). A molecular formula of C_34_H_38_N_4_O_8_ was deduced for *Mc*-NCC-49 by mass spectrometry. The molecular constitution of *Mc*-NCC-49 was again elucidated with the help of heteronuclear NMR spectroscopic investigations, which indicated it to be a 8^2^-hydroxy-13^2^-carboxyl-3^1^,3^2^-didehydro-1,4,5,10,15,20,24-octahydro-4,5-seco-(22 H,24 H)-phytoporphyrin. The presence of one hydroxyl group at the side chain C8 (of the tetrapyrrolic NCC core), and of two carboxyl groups elsewhere, indicated *Mc*-NCC-49 (see Figure 3] and Table 2) to have the same molecular constitution as NCCs isolated from senescent leaves of oil seed rape (*Brassica napus*),[33] of *Arabidopsis thaliana,*[34] or of spinach (and then named *So*-NCC-3).[35]

A still less polar NCC, *Mc*-NCC-59, was a minor fraction in most extracts from peels of yellow bananas. Mass spectra revealed *Mc*-NCC-59 to have the molecular formula of C_41_H_50_N_4_O_13_, that is, to be more complex than the other *Mc*-NCCs, listed above. The molecular constitution of *Mc*-NCC-59 was derived tentatively as a 8^2^-β-glucopyranosyl-13^2^-methoxycarbonyl-3^1^,3^2^-didehydro-1,4,5,10,15,20,24-octahydro-4,5-seco-(22 H,24 H)-phytoporphyrin. *Mc*-NCC-59 would thus carry a hexose moiety (presumably a glucopyranose unit) at the hydroxyl group at the side-chain C8 of the tetrapyrrolic NCC core, as observed in other NCCs[9] (see Table 2). *Mc*-NCC-59 is thus suggested to have the same molecular constitution as NCCs isolated from senescent leaves of tobacco (*Nicotiana rustica*)[36] or of maize (*Zea mais*) (then named *Zm*-NCC-2)[37] (see Figure 3] and Table 2).

The least polar NCC, *Mc*-NCC-61, was a minor fraction in the extracts of yellow banana peels (0.47 μmol (302 μg) from 99 bananas). Mass spectrometry revealed *Mc*-NCC-61 to have the molecular formula C_35_H_40_N_4_O_8_. The molecular constitution of *Mc*-NCC-61 was elucidated with the help of heteronuclear NMR spectroscopic investigations, which revealed it as a 8^2^-hydroxyl-13^2^-methoxycarbonyl-3^1^,3^2^-didehydro-1,4,5,10,15,20,24-octahydro-4,5-seco-(22 H,24 H)-phytoporphyrin. *Mc*-NCC-61 is thus an isomer of the fluorescent catabolite *Mc*-FCC-62 (see above), and it has the same molecular constitution as NCCs analyzed previously,[9] for example, *Cj*-NCC-1 from senescent leaves of the Katsura tree (*Cercidiphyllum japonicum*),[38,39] or of *So*-NCC-4 from spinach (*Spinacia oleracea*)[35] (see Figure 3 and Table 2).

Two further (very minor) polar components having the UV-spectral signature of NCCs were collected, which were named *Mc*-NCC-55 and *Mc*-NCC-58 (see Figure 2). These NCCs were identified by HPLC with the two main NCCs obtained from acid-induced isomerization of the *hm*FCC *Mc*-FCC-56 (see below and the Supporting Information, Figure S2–S5).

**Slow acid-induced-isomerization of** ***Mc*****-FCC-56 to** ***Mc*****-NCC-55 and** ***Mc*****-NCC-58**: In an analytical experiment, a sample of raw *Mc*-FCC-56 (containing about 30 % of its isomer *Mc*-FCC-53) was stored in an aqueous solution acidified to a pH of about 4, and was analyzed by HPLC. After 24 h about half of the *hm*FCC had converted; after 70 h complete conversion to two major NCCs was observed (see the Supporting Information, Figure S2). In a preparative experiment, a solution of *Mc*-FCC-56 (31 μmol; containing about 30 % of its isomer, *Mc*-FCC-53) in aqueous citric acid (0.02 m) was stored for 159 h under an argon atmosphere in the dark at 15 °C. Analysis of the resulting solution by HPLC indicated disappearance of FCCs and appearance of two major NCCs, which were isolated by preparative HPLC. The UV spectra of the two compounds confirmed them to be NCCs (see the Supporting Information, Figure S3), which were characterized as isomers of *Mc*-FCC-56 by their ESI-MS. They were further identified with *Mc*-NCC-58 and *Mc*-NCC-55, two fractions occurring naturally in extracts of banana peels (see Figure 2] and the Supporting Information, Figures S4 and S5). The less polar and more abundant *Mc*-NCC-58 exhibited a CD spectrum typical of the known natural NCCs.[39,40] The CD -spectrum of *Mc*-NCC-55 had nearly mirror-image properties to that of *Mc*-NCC-58 (see Figure 4] and the Experimental Section), indicating these two isomeric NCC to be C15 epimers.[40]

**4:**
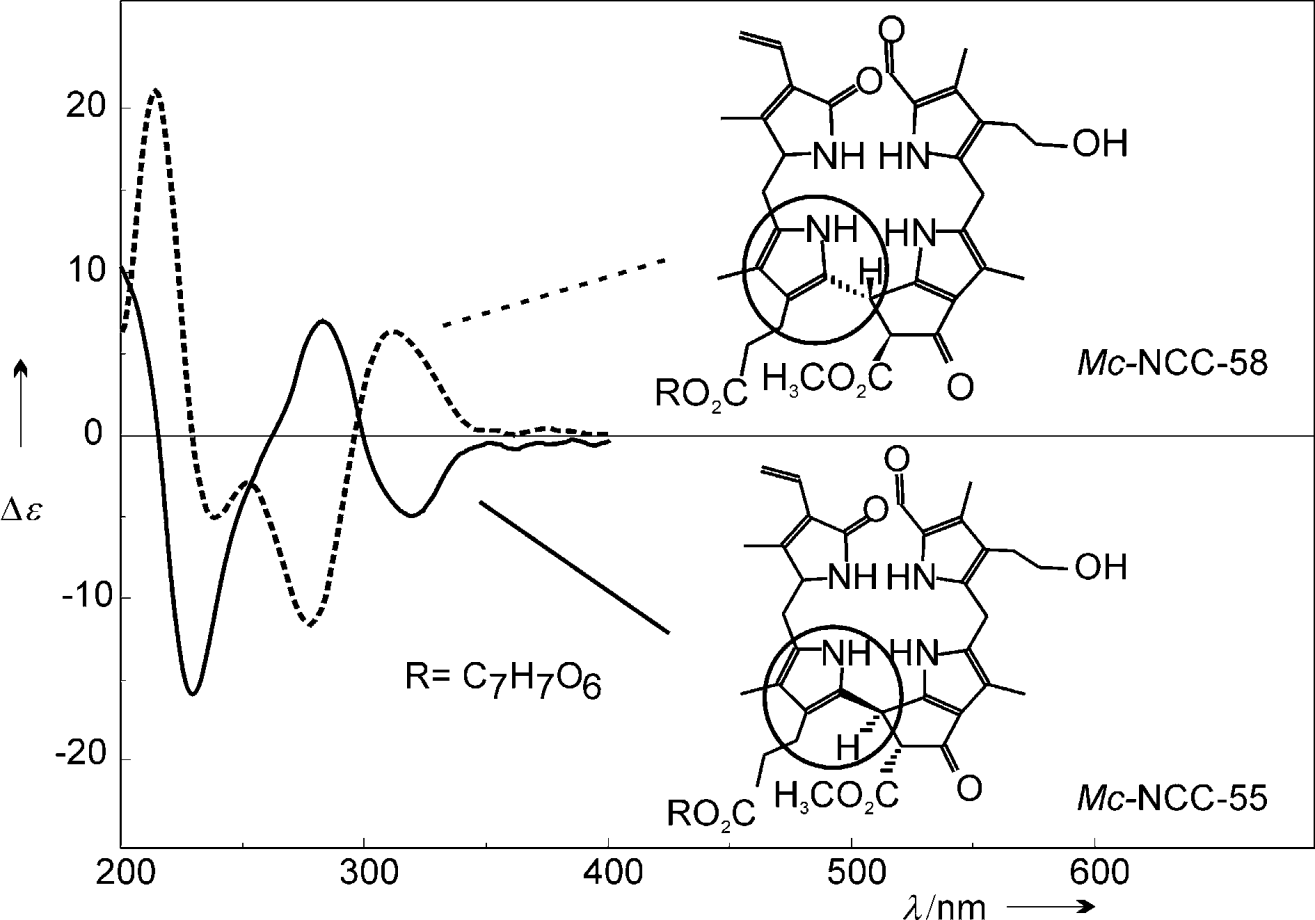
CD spectra of *Mc*-NCC-55 (solid line) and *Mc*-NCC-58 (dashed line) in MeOH/potassium phosphate buffer (100 mm), pH 7, ≍1/1 (v/v). Samples of *Mc*-NCC-55 and *Mc*-NCC-58 were obtained from isomerization of *Mc*-FCC-56 induced by treatment with dilute aqueous citric acid (see the Experimental Section and Supporting Information for more details).

**Identification of** ***Mc*****-NCC-42 with an NCC from spinach leaves**: *Mc*-NCC-42 was deduced to have the same molecular constitution as *Hv*-NCC-1[8] and as *So*-NCC-2.[32] The latter two NCCs were assigned earlier to be (C1)-epimers, derived from *p*FCC[29] or from *epi-p*FCC, respectively.[30,41] The pertinent NMR-data of *Mc*-NCC-42 were practically identical to those of *So*-NCC-2.[32] Nevertheless, the identity of *Mc*-NCC-42 with either *Hv*-NCC-1 or *So*-NCC-2 was further tested by HPLC. Solutions of the three NCCs were separately analyzed by HPLC, as were 1:1 (v/v) mixtures of the solutions of *Mc*-NCC-42 with *Hv*-NCC-1, and with *So*-NCC-2, respectively (see the Supporting Information, Figure S6). The HPL-chromatographic behavior of *Mc*-NCC-42 and of *So*-NCC-2 was the same, whereas that of *Mc*-NCC-42 and of *Hv*-NCC-1 proved to be different.

**Profile of abundance of natural FCCs during ripening of** ***Mc*****-bananas**: When bananas were bought at the early ripening stage (rs) 5 *Mc*-FCC-62 and *Mc*-FCC-56 represented the major FCC fractions observed in their peels, whereas the more polar *Mc*-FCC-53 was less abundant. On the following day, quantification of the major *Mc*-FCCs in the peels of the now fully yellow ripe bananas (i.e., at rs=6–7[28) indicated a higher total amount of the FCCs, but a significant decrease of the amount of *Mc*-FCC-62 (see Figure 5). In parallel, both of the esterified *hm*FCCs (*Mc*-FCC-56 and *Mc*-FCC-53) became more abundant. Further ripening through days two to four led to practically a complete disappearance of *Mc*-FCC-62. During the same time, the amount of *Mc*-FCC-56 also decreased, whereas, on day 2 and 3 (at rs=7–8, when the formation of "senescence-associated" dark spots set in), the more polar and more highly functionalized *Mc*-FCC-53 underwent an increase, to be followed by a decrease on days three and four.

**5:**
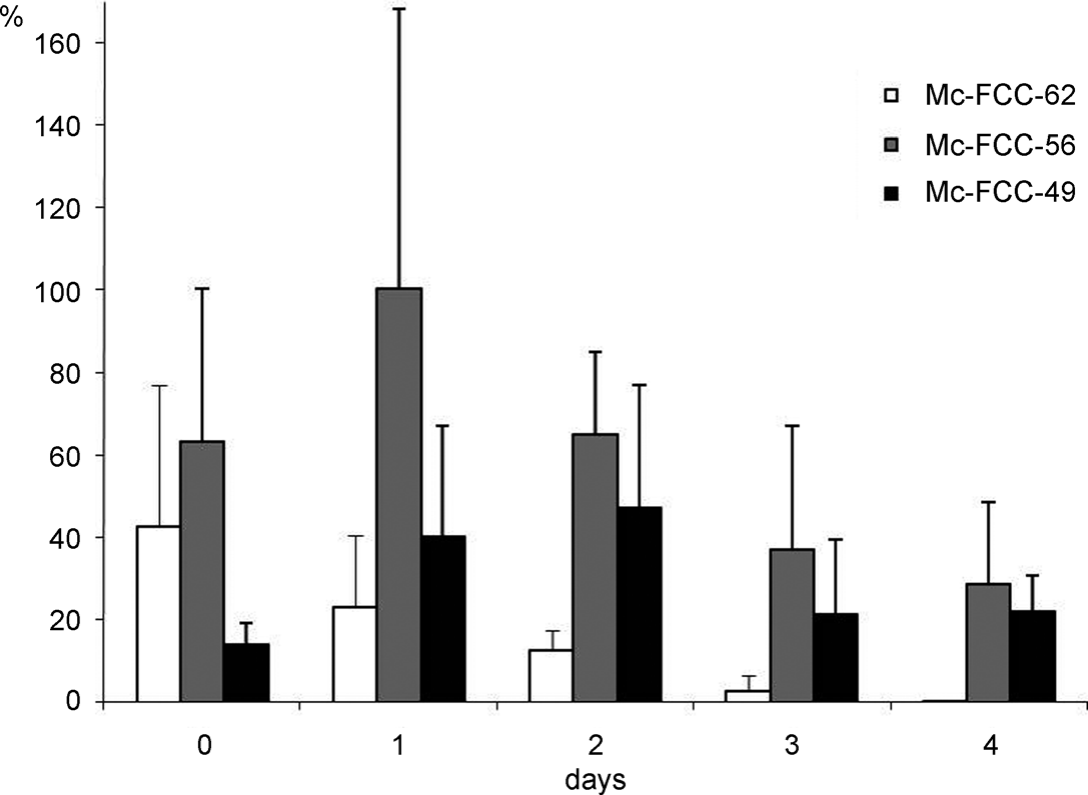
Profile of abundance of *Mc*-FCCs during ripening of bananas. The relative amounts of three main *Mc*-FCCs in peels of ripening bananas are depicted as a function of the time of storage at room temperature, beginning with the day of their acquisition (day 0, at ripening stage (rs)=5) and analyzed on the following four days (senescence-associated dark spots typically appear on days 3 and 4); FCC fractions were extracted from 3–5 banana peels at the indicated days; for quantitative analysis, the peak areas of the three FCCs in the fluorescence traces of HPL-chromatograms were determined individually; the averaged data given here was obtained by dividing the total area by the number of determinations.

**Light- and fluorescence-microscopic analyses of green and de-greened banana peels**: Surface sections of banana peels of different stages of ripeness (see the Supporting Information, Figure S8) showed that green bananas (Figure S8 A), bright yellow bananas (rs=5–6)[28] (Figure S8 B), ripe bananas with few brown spots (rs=7–8) (Figure S8 C), and overripe bananas (rs=10–11) (Figure S8 D) still retained intact chloroplasts in the guard cells of the stomata (Figure S8 E–L), as visualized by their red fluorescence seen in the confocal laser-scanning microscopic images (Figure S8 I–L). Remarkably, in the yellow sections of banana peels at the late-ripening stages (rs 6–9), the chloroplasts in the guard cells appeared to be still intact. Only in the dark brown areas the epidermal and respective guard cells were dead, and red fluorescence was much less pronounced or absent, as observed earlier.[22]

When investigating sub-epidermal parenchyma cells in surface sections in a similar way, a marked difference between the green and yellow peels was found (see Supporting Information, Figure S9). Whereas green peels exhibited intact green chloroplasts (Figure S9 A), which showed bright red fluorescence (Figure S9B), only small, yellowish gerontoplasts were observed in de-greened sub-epidermal parenchyma cells of yellow banana peels (at rs=6) (Figure S9 D). The red fluorescence was totally lost in these cells (Figure S9 E). Instead, blue fluorescence increased in the cell lumen of parenchyma cells in de-greened peels (Figure S9 F), which is typical for FCCs. In contrast, green-banana peels showed less blue fluorescence in the cell lumen of sub-epidermal parenchyma cells, whereas the cell walls retained their fluorescence (Figure S9 C). The cells from peels of overripe bananas had a similar appearance as those of yellow ones (not shown). However, their brown areas contained dead cells.[22]

## Discussion

The change of color of sweet bananas (*Musa acuminata*; short: *Mc*-bananas) from green to yellow is a well-known visual sign of their ripening. Indeed, color is an important criterion for fruit ripeness and quality,[42] and the molecular mechanisms responsible for its generation are a subject of obvious interest (see, for example, ref. [43). Surprisingly, the ease of the observation of the color change, due to disappearance of chlorophyll in bananas (and in many other important fruits), contrasts with a lack of knowledge of the chlorophyll catabolites produced in fruits.

Studies of chlorophyll breakdown in senescent leaves have suggested a common, metabolically controlled and directed path that yields colorless NCCs as its typical tetrapyrrolic (end) products (see Figure 1).[1,13,31] This pathway of chlorophyll catabolism passes through the stage of pheophorbide a to give the colorless "primary" FCCs (either *p*FCC, or its epimer, *epi*-*p*FCC) in the chloroplasts ("gerontoplasts") as products of the "early" phase of chlorophyll breakdown.[2,31] The original structural analysis and identification of these short-lived (and often elusive) FCCs were only possible with the help of ex-vivo preparations using enzyme active extracts from senescent leaves.[29,30] Indeed, in weakly acidic aqueous solutions, FCCs with a free propionic acid substituent undergo a fast and thermodynamically favorable isomerization to the corresponding stable NCCs.[39,40] This type of chemical isomerization is now seen as accounting for the natural formation of the NCCs in the vacuoles, as the "last step" of chlorophyll breakdown.[13,39] Natural NCCs carry a variety of polar peripheral functional groups,[9] which are thus presumed to reflect the corresponding modifications of their elusive FCC-precursors.[2] Indeed, the structures of FCCs are mostly inferred from those of the corresponding NCCs, which often accumulate in senescent leaves (see Figure 3] and Tables 1 and 2).[13,14]

Chlorophyll breakdown in leaves has, primarily, been rationalized as a kind of detoxification process:[14] It rapidly, and directly, degrades the potentially phototoxic chlorophylls to colorless (and "photo-inactive") tetrapyrroles[44] and, thus, removes chlorophylls from their protein partners, which are then made accessible to proteolysis.[45] Hence, chlorophyll breakdown contributes indirectly to the controlled recuperation of nutrients, of reduced nitrogen, in particular,[46] and also helps extending the viability of senescent cells before the leaves of deciduous plants are shed.

In contrast, degradation of chlorophyll in ripening fruit has so far been less well studied, and remains a largely puzzling phenomenon. In some fruits, such as apples and pears, chlorophyll catabolism yields colorless NCCs, following the pathway found in senescent leaves.[5] Obviously, the natural disappearance of chlorophyll in ripening fruit eliminates their capacity for photosynthesis, with stringent consequences for metabolism.[42] However, chlorophyll breakdown may also play a role within the peel of the fruit, similar to that deduced in senescent leaves.[46] Indeed, chlorophyll breakdown in peels of ripening bananas does not only provide NCCs, it leads also to an unprecedented accumulation and structural variety of "persistent" *hm*FCCs. As such, the disappearance of the chlorophylls in ripening fruits induces an easily observable change of color, and provides an optical signal relevant for the interaction with frugivors.[47] Thus, chlorophyll breakdown in ripening fruits serves the communication of fruit-producing plants with (frugivorous) animals in their environment.

**Structures of chlorophyll catabolites in extracts of ripening banana peels**: Using HPLC, with on-line analysis by UV/Vis absorbance and fluorescence spectra, chlorophyll catabolites were systematically detected in the extracts, classified as FCCs and NCCs, and specified individually according to their polarity, for example, as *Mc*-NCC-*t*_R_. The structures of five *Mc*-FCCs and of seven *Mc*-NCCs were elucidated with mass spectrometric[48–50] and NMR spectroscopic analyses,[51,52] mainly (see the Results section). A sixth FCC (tentatively named *Mc*-FCC-71) was shown to be a likely isolation artifact (see the Results section and the Supporting Information). The present analysis of chlorophyll catabolites in bananas establishes a firm structural basis for further detailed glimpses into the biochemistry of chlorophyll breakdown.

Recently, we described four of the fluorescent *Mc*-FCCs occurring in extracts of banana peels.[21,22] These were classified as the "hyper-modified" *Mc*-FCC-56 and *Mc*-FCC-49, as well as their less abundant isomers, named *Mc*-FCC-46 and *Mc*-FCC-53. In contrast to the (few) natural FCCs identified earlier,[13] "hyper-modified" FCCs (*hm*FCCs) carry an intriguing ester function at the propionate side chain.[21,22] Such ester functions stabilize *hm*FCCs against their spontaneous acid catalyzed isomerization to NCCs and render them "persistent".[22,40] As shown here, a further natural FCC of medium polarity was present in *Mc*-bananas during early stages of ripening (at rs=5),[28] in addition to the four "persistent" *hm*FCCs.[21,22] This FCC was named *Mc*-FCC-62, and its structure was established. *Mc*-FCC-62 carries a free propionic acid function, as is characteristic of most natural FCCs. It is prone to conversion to an isomeric NCC, which was identified in bananas as *Mc*-NCC-61. Compared with "primary" FCCs, *Mc*-FCC-62 exhibited an additional hydroxyl modification at the ethyl side-chain. This is the indirectly deduced earliest modification of FCCs in a variety of senescent leaves[13] and in fruit.[20,53] *Mc*-FCC-62 (8^2^-OH-*epi-p*FCC) (see Figure 3) may therefore be classified as an unprecedented example of a prototypical "secondary" FCC (*s*FCC). Inspection of the time dependence of the accumulation of the different major FCCs in the peels of ripening *Mc*-bananas indicated the amount of *Mc*-FCC-62 to be highest in freshly bought *Mc*-bananas, which were generally acquired at rs=5 (see the Results section and Figure 5). The content of *Mc*-FCC-62 decreased rapidly during the first two days of storage at room temperature (i.e., at rs=6–7), compensated, however, by a corresponding simultaneous increase of the amounts of the *hm*FCCs, *Mc*-FCC-53 and *Mc*-FCC-56. Indeed, the total content of FCCs was maximal in bright-yellow bananas (at rs=6), that is, typically on "day two" after acquisition.[21] These findings and the structures of the *hm*FCCs suggested a biosynthetic sequence, in which *Mc*-FCC-62 would be their precursor.[22]

The structures of the major five (of the seven) *Mc*-NCCs identified, so far (*Mc*-NCC-26, *Mc*-NCC-42, *Mc*-NCC-49, *Mc*-NCC-59 and *Mc*-NCC-61, see Table 2), suggested a second biosynthetic path, that also involved *Mc*-FCC-62 as their precursor. Indeed, these five NCCs carried a free propionic acid function and appear to be all formed through elusive *m*FCCs. Our earlier studies of chlorophyll breakdown in senescent leaves of other plants, such as spinach (*Spinacia oleracea*),[32,35] oil seed rape (*Brassica napus*),[33] or *Arabidopsis thaliana*[34] and so on, has provided precedence for NCCs with the molecular constitution of the *Mc*-NCCs. The structures of the *Mc-*NCCs suggest the intermediary existence of the corresponding *m*FCCs (*Mc*-FCCs, see Figure 3). In analogy to the related findings in *Arabidopsis*,[34] the lack of observation of the *m*FCCs is assumed to be due to their rapid spontaneous isomerization to the corresponding *Mc*-NCCs, once these FCCs have reached the vacuoles.[2]

Two further, minor NCC fractions (*Mc*-NCC-55 and *Mc*-NCC-58) were tentatively identified by HPLC-analysis. They co-eluted with "authentic" samples of *Mc*-NCC-55 and *Mc*-NCC-58 obtained by acid-induced isomerization of *Mc*-FCC-56. These two minor NCC fractions thus represent NCCs with an unprecedented propionate ester function. Remarkably, the CD spectrum of one of them (of *Mc*-NCC-55) was a near mirror image of the CD spectra of the known NCCs, indicating an opposite absolute configuration at C15,[40] which is consistent with their hypothetical formation from *hm*FCCs, such as *Mc-*FCC-56, by a stereo-unspecific isomerization.

**Chlorophyll breakdown in** ***Mc*****-bananas involves** ***epi-p*****FCC and the reductase RCCR-2**: Two stereo-divergent classes of the critical enzymes, called "red chlorophyll catabolite" reductases (RCCRs), exist in higher plants, typified as class-1 or class-2 RCCRs.[13] This surprising variability of chlorophyll breakdown appears to reflect a late outcome of their evolution,[41] and was discovered when the first structures of FCCs were elucidated. These turned out to be (C1)-epimeric "primary" FCCs, named *p*FCC and *epi*-*p*FCC.[29,30] Indeed, in the context of a purely catabolic path, the stereo-structure of a presumed "detoxification" product appeared of lesser (biological) relevance.[30] A representative of the class-1 RCCRs was expressed from *Arabidopsis,*[54] and its crystal structure was deduced recently.[55,56]

The type of RCC-reductase active in *Mc*-bananas was deduced as a class-2 RCCR,[41] based on the structural and chromatographic characteristics of the polar *Mc*-NCC-42. This banana NCC was identified with *So*-NCC-2, an NCC from spinach leaves (*Spinacia oleracea*)[32] (see the Supporting Information, Figure S5). By the same type of experiments, *Hv*-NCC-1 was shown to differ from *Mc*-NCC-42. Identification of *Mc*-NCC-42 with *So*-NCC-2 indicated the *Mc*-NCCs to originate from *Mc-*FCCs that belong to the stereo-lineage of *epi*-*p*FCC, which is formed by a "class-2" RCCR. The (stereochemical) identification of *Mc*-NCC-42 with *So*-NCC-2 implies also pairwise identities of *Mc*-NCC-26 and *So*-NCC-1, of *Mc*-NCC-49 and *So*-NCC-3, as well as of *Mc*-NCC-61 and *So*-NCC-4.

**Structures of chlorophyll catabolites reveal a novel split path of chlorophyll breakdown in** ***Mc*****-bananas and** ***s*****FCC as the branching point and as a "last common precursor" of the downstream catabolites**: Based on the information on the structures and on the abundance of colorless-chlorophyll catabolites, chlorophyll breakdown in the peels of ripening banana unfolds in a remarkable fashion. It is consistent with a "common earlier" path that provides *epi*-*p*FCC as the short-lived "primary" FCC.[13] However, no evidence was found, neither for (green) type I breakdown products[23] nor for catabolites derived from them.[17] The specific accumulation of *Mc*-FCC-62 at the stage of still incomplete ripening (classified as rs=5)[28] is a remarkable feature of chlorophyll breakdown in the peels of *Mc*-bananas. The deduced structure of *Mc*-FCC-62 reflects the first modification of the (hypothetical) *epi*-*p*FCC[30] by hydroxylation at the terminal carbon of the ethyl group at C8. Indeed, the characteristic presence of such a hydroxylation among the peripheral modifications in most NCCs, found to date, suggest it to be a direct natural modification of "primary" FCCs.[13] All *Mc*-NCCs, and all *Mc*-FCCs, including the "hyper-modified" FCCs (*hm*FCCs), carry an oxygen-functionality at the terminal carbon of the substituent at C8, which may be modified by further functionalization. *Mc*-FCC-62 represents, therefore, the first example of a completely characterized "secondary" FCC (*s*FCC).

From the structures of the chlorophyll breakdown products in the peels of *Mc*-bananas, *Mc*-FCC-62 was indicated to be a rational biosynthetic precursor and (actually) the "last common precursor" of all *Mc-*FCCs and *Mc*-NCCs appearing further "downstream" in the path. The abundance profiles of the natural chlorophyll catabolites in ripening *Mc*-bananas were also compatible with this role of *Mc*-FCC-62, which was abundant only at the early ripening stages (up to rs=5), and disappeared within about two days of further ripening. During this time the more polar *hm*FCCs, *Mc*-FCC-56 and *Mc*-FCC-53, became the dominant fractions, compatible with their suggested appearance as products of further functionalization of *Mc*-FCC-62 by catabolic enzymes[22] (see Figure 5). *Mc*-FCC-62 thus also marks the branching point of two major processing lines of chlorophyll breakdown in the banana peels, as derived on structure-based considerations (see Figure 3): In one branch, *Mc*-FCC-62 gives rise to the observed "hyper-modified" *Mc*-FCCs. In the other, this *s*FCC serves as the precursor of the *m*FCCs. The existence of these elusive catabolites is deduced from the detection of their presumed isomerization products, the correspondingly modified *Mc*-NCCs.

The early steps of chlorophyll breakdown thus yield FCCs and still occur in the chloroplasts.[13,14] However, NCCs, the typical "final" catabolites, are found in the vacuoles.[31,57] This is a consequence of transport processes that take place during chlorophyll breakdown. These processes, as well as the location of the catabolic enzymes and the corresponding spatial (re)distribution of chlorophyll catabolites during ripening and senescence are intriguing topics. Most studies in this area, if not all, have concentrated on chlorophyll breakdown in senescent leaves.[2] In analogy, the "earlier stages" of chlorophyll breakdown in ripening fruit are assumed to operate within the chloroplast (see Figure 6). They are presumed to follow the "common path" via pheophorbide a and an enzyme-bound form of the red chlorophyll catabolite (RCC) to provide short-lived "primary" FCCs, as the first colorless catabolites.[13,14] In the banana peels, the "primary" FCC escaped its observation and was deduced, indirectly, to be *epi-p*FCC (see Figure 3). However, an unprecedented accumulation of a prototypical "secondary" FCC (*s*FCC), the hydroxylated *Mc*-FCC-62, was seen at an early stage of ripening of bananas (at rs=5).[28] Its formation in ripening bananas represents the likely result of an as yet unidentified hydroxylating enzyme[44] that introduces the indicated first modification of *epi*-*p*FCC (see the Experimental Section, Figure 8).

**6:**
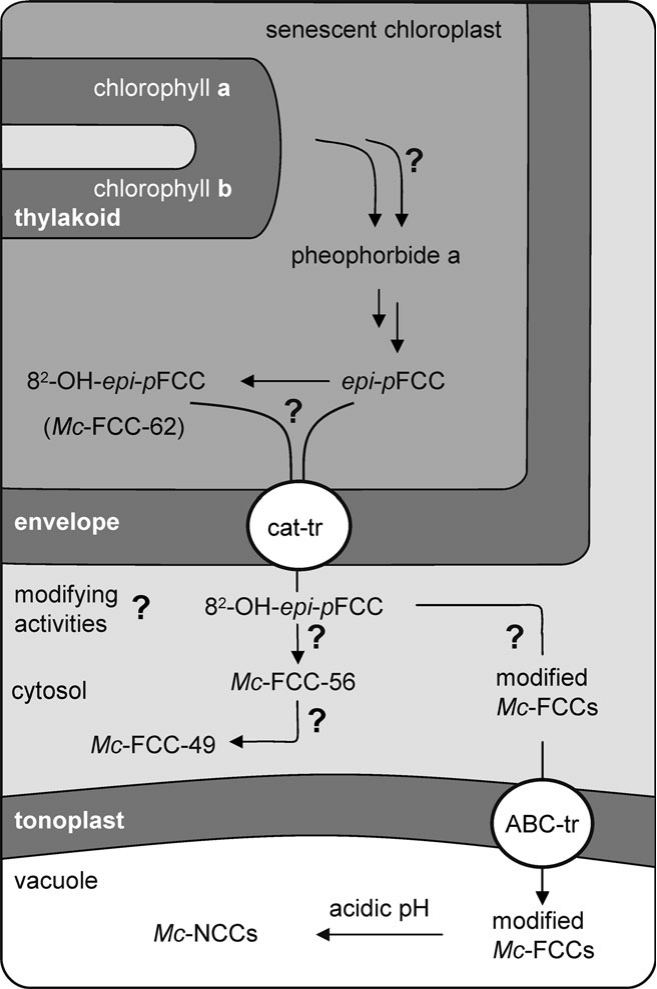
Structure-based topographical model of a hypothetical split path of chlorophyll breakdown in the peels of bananas. Chlorophylls are degraded by chloroplast enzymes to *epi*-*p*FCC (and possibly to the *s*FCC, 8^2^-OH-*epi*-*p*FCC). *Epi*-*p*FCC (and/or 8^2^-OH-epi-*p*FCC) are transported via the envelope into the cytosol (with assistance of a "catabolite" transporter, cat-tr). In the cytosol, 8^2^-OH-*epi-p*FCC is converted to elusive modified FCCs (*m*FCCs), which are transported by a tonoplast-bound ABC-transporter (ABC-tr) into the vacuole. There, *m*FCCs isomerize to the *Mc*-NCCs. Alternatively, a hypothetical cytosolic enzymatic esterification converts 8^2^-OH-*epi*-*p*FCC into *Mc*-FCC-56; subsequently, *Mc*-FCC-56 is likely to be converted to *Mc*-FCC-49 by the action of a β-glucosylating enzyme.

Similar to the situation suggested for the well-studied chlorophyll breakdown in senescent leaves,[2] most, if not all of the further modifications of the FCCs, which give *m*FCCs in the banana peels, would be presumed to occur in the cytosol. This scenario requires export of *Mc*-FCC-62 (or of *epi-p*FCC) across the envelope of the chloroplasts, which is thought to occur by a catabolite transporter (cat-tr) and to be activated by ATP[44] The various elusive *m*FCCs would be formed by one, or several, enzyme-catalyzed transformations of *Mc*-FCC-62, for example, by hydrolysis of its methyl ester function,[12] by oxidative dihydroxylation of its vinyl group, or by glucosylation of its hydroxyl function. These *m*FCCs would then be transported from the cytosol through the tonoplast by an apparently efficient, but rather substrate-unspecific ABC transporter (ABC-tr) into the (acidic) vacuoles, where a rapid FCC to NCC isomerization takes place (see Figure 6).[14]

A second and alternative branch of chlorophyll breakdown yields the "persistent" *hm*FCCs in a hypothetical parallel esterification of *Mc*-FCC-62. The accumulation of *Mc*-FCC-62 and of *hm*FCCs at early stages of ripening (at rs=5 and 6),[28] as well as the deduced rapid formation of the latter from the former, suggest the temporal co-existence of *s*FCC and of *hm*FCCs in a common compartment. Presumably, this is the cytosol, and not the vacuole, as in the latter *Mc*-FCC-62 would be assumed to disappear very rapidly and to isomerize to the corresponding NCC, *Mc*-NCC-61. Two competing pathways are, thus, suggested to operate in the cytosol, and to be catalyzed by enzymes that remain to be identified. The accumulation of *hm*FCCs in ripening bananas appears to be consistent also with their main localization in the cytosol.

However, the further fate of the *hm*FCC and their possible eventual relocation are unknown. *hm*FCCs have been suggested to be remarkably persistent under physiological conditions. To test this, the behavior of *Mc*-FCC-56 was examined in a homogeneous acidic medium. Slow isomerization to two major NCCs (*Mc*-NCC-55 and *Mc*-NCC-58) was seen (see the Supporting Information, Figure S3). The acid-catalyzed isomerization of *Mc*-FCC-56 at pH 4 was slower by about a factor of 50 than that of *epi*-*p*FCC at pH 4.9,[39] thus verifying the expected lesser tendency of *hm*FCCs to undergo a conversion to NCCs. The less polar and more abundant *Mc*-NCC-58 exhibited a CD spectrum (see Figure 4) typical of the known natural NCCs.[40] The quasi mirror image of the CD spectrum of *Mc*-NCC-55 indicated it to be an atypical C15 epimer.[40] The acid-induced isomerization of *Mc*-FCC-56 thus also lacked stereoselectivity, with a preference of only about 2:1 for the "natural" epimer, which is consistent with earlier findings with synthetic model compounds.[40]

**Hyper-modified FCCs are a persistent source of blue fluorescence**: Chlorophyll breakdown in the peels of ripening bananas provides "hyper-modified" FCCs (*hm*FCCs), in a newly revealed branch of the degradation pathway.[21,22] The naturally accumulating *hm*FCCs induce (the peels of) ripening bananas to fluoresce blue when excited by UV light. These luminescent products of chlorophyll breakdown may, thus, serve as a specific communication with the exterior world. The luminescence of ripe bananas can be recognized by animals sensitive to that section of the light spectrum, and such bananas are likely to appear more attractive to them. Human eyes may also perceive FCCs in bananas, but, typically, only as optical brighteners.

*hm*FCCs appear to be generated in the cytosol from less polar precursor-FCCs. The known further fate of *Mc*-FCC-56 is associated with the formation of the more complex, and more polar*, Mc*-FCC-49 during later stages of fruit ripening, and accumulation of the latter in blue, luminescent rings[22] that surround the "senescence associated"[58] dark spots. The cytosol, the presumed predominant location of the formation of the *hm*FCCs, represents an extended compartment in the plant cell that may also function as a well-accessible site for their eventual intercellular transport. Indeed, the metabolic investment in the unique biosynthesis of these highly modified *hm*FCCs suggests the consideration of a physiological role (see below).

Accumulation of *hm*FCCs in commercially available *Mc*-bananas has been a stunning finding. It induced additional studies, which showed that the formation of *hm*FCCs is not a consequence of artificial ripening of this climacteric fruit by treatment with ethylene gas. Exploratory studies with naturally ripened *Mc*-bananas from a plantation on the Island of Tenerife, Spain, indicated these bananas to contain a distribution of the dominating *hm*FCCs, very similar to that of ethylene-ripened commercial ones (see the Supporting Information, Figure S7). However, differences in the content of NCCs, the minor type of chlorophyll catabolites in the banana peels, were noted, requiring further studies to test their significance.

In-vivo light- and fluorescence microscopic studies with *Mc*-bananas revealed the intense blue fluorescence of FCCs to predominantly occur in sub-epidermal parenchyma cells of bright yellow banana peels (see the Supporting Information, Figure S9). A temporarily marked increase of this type of fluorescence has been described in areas around the growing "senescence-associated dark spots".[22] However, the fluorescence of cell-wall components (such as phenols, lignin, and coumaric acid derivatives) may also contribute to part of the overall "blue" luminescence.[59]

In de-greened sub-epidermal parenchyma cells, a characteristic rearrangement of the chloroplasts takes place. Whereas early studies described a "chromoplast" differentiation,[60] the remaining plastids are characterized by thylakoid membrane loss and occasional accumulation of lipid globules, and are now classified as gerontoplasts.[61,62] However, on the largely simultaneously de-greening peels of banana, chlorophyll breakdown does not occur (in a synchronized fashion) in all cells: in fully ripe bananas, the guard cells of stomata still contain intact chloroplasts and appear to be functional (see the Supporting Information, Figure S8). The observed "delay" of the senescence of stomatal guard cells of the banana peels is similar to the one in senescent leaves, in which the process of chloroplast aging in stomata has been shown to lag behind the one of the parenchyma cells.[63] This underscores the temporarily local relevance of photosynthesis at this developmental stage, which provides the metabolic support in the guard cells, to achieve the required continuous control of the exchange of oxygen, water, and other volatiles with the environment.[64] As one physiological aspect, oxygen-exchange remains important for respiration, because mitochondria are more stable than chloroplasts. When stomata eventually undergo a retarded form of chlorophyll breakdown in over-ripe bananas, their loss of function, and death, is marked by the appearance of "senescence-associated" dark spots,[22] which eventually coalesce to give a dark and leathery skin texture.

**On the physiological roles of tetrapyrrolic chlorophyll catabolites in higher plants**: In senescent leaves of deciduous trees chlorophyll breakdown occurs rapidly in the largely synchronized pattern characteristic of the fall colors, and appears mainly to yield a number of NCCs as the "final" tetrapyrrolic products.[9,13] In senescing leaves of two evergreens (Peace Lily (*Spathiphyllum wallisii*)[65] and banana (*Musa acuminata*)[66), chlorophyll breakdown has been revealed to be more complex. Only a few ripening fruits have been analyzed, so far, for their non-green chlorophyll catabolites.[67] In apples and pears, only known NCCs were detected.[5,20] As delineated here in the peels of ripening bananas, a diverse set of non-green chlorophyll catabolites were discovered: unique "persistent" *hm*FCCs, and a variety of typical NCCs. The structures of these chlorophyll catabolites indicated a forked pathway of chlorophyll breakdown in this fruit that splits into two separate "downstream" lines.

Accumulation of *hm*FCCs and the existence of a split path of chlorophyll breakdown are, thus, not unique for ripening banana fruits, but are rather a more general outcome of chlorophyll catabolism in higher plants. The complex structures of the ubiquitous degradation products of chlorophyll, and the hypothetical metabolism that generates them, suggest such chlorophyll catabolites to be more than mere products of a detoxification process, and may point to a physiological role of tetrapyrrolic chlorophyll catabolites in senescent leaves and in ripening fruit. Indeed, the tetrapyrrolic chlorophyll catabolites are remarkably similar, structurally, to important plant pigments, such as the bilins,[68,69] which are products of the natural breakdown of heme.[70] As NCCs are effective antioxidants,[20] their availability may be helpful in extending the viability of the tissue during leaf senescence or fruit ripening. The structurally related FCCs may have a range of comparable chemical properties, which, as yet, have hardly been investigated. Therefore the abundance and the expected unusual properties of such tetrapyrrolic chlorophyll catabolites call for further investigations of their biologically relevant chemical properties, as well as of their possible physiological relevance in higher plants.

Fluorescent chlorophyll catabolites, such as the hyper-modified FCCs, also stand out on account of their optical properties:[22] The blue luminescence of "persistent" *hm*FCCs in fruits may signal ripeness to frugivorous animals. The luminescence of intact bananas can also be monitored non-invasively by humans: Sensitive (time-, space-, and wavelength-resolved) luminescence measurements may make use of the natural formation of FCCs as fluorescent markers.[53] In banana peels, *hm*FCCs accumulate in freshly ripening tissue and in still viable, senescent cells in the transition region from intact, ripened to dead peel tissue. Persistent fluorescent chlorophyll catabolites, which are endogenously provided by (one branch of the split path of) chlorophyll breakdown, may, hence, commend themselves as natural molecular *in-vivo* reporters of senescence and senescence-associated cell-death symptoms.

## Experimental Section

**Materials**

*Plant material*: Freshly ripe bananas (*Musa acuminata*, Cavendish cultivar) were bought from different stores in Innsbruck at the early ripening stage rs=5 (see[20,21); bananas of the cultivar *Musa cavendish* at different stages of ripening (unripe (rs <4), freshly ripe (rs=5) and over-ripe (rs=8–9)[28] were also harvested from the plantation Malpais Trece (Garachico, Island of Tenerife, Spain) and were transported at ambient temperature to Innsbruck, where they were directly analyzed.

*Chemicals*: Commercially available solvents (reagent-grade) were redistilled before use for extractions. HPLC grade methanol (MeOH) was from Merck (Darmstadt, Germany) and Acros Organics (Geel, Belgium); acetonitrile (ACN) from Acros Organics (Geel, Belgium); formic acid, purum (HCO_2_H), potassium dihydrogen phosphate puriss. p. a., and potassium phosphate dibasic-anhydrous puriss.p.a. were from Fluka (Buchs, Switzerland). Amberlite IRA-900, Cl-form ion exchange resin was from Acros Organics (Geel, Belgium). Sep-Pak-C18 Cartridges were from Waters Associates.

**Methods**

*HPL-Chromatographic methods*: Solvent: A: 100 mM potassium phosphate (pH 7.0); B: MeOH; C: water (0.002 % HCO_2_H); D: ACN (0.002 % HCO_2_H).

*Analytical HPLC:* Dionex Summit HPLC system, used with manual sampler, P680 pump, online degasser and diode array detector and Jasco FP-920 fluorescence detector. Injection loop 1 mL (Rheodyne injection valve), a Hypersil ODS 5 μm 250×4.6 mm i.d. column, used at room temperature, Phenomenex ODS4 mm x 3 mm i.d. pre-column; flow rate 0.5 mL min^−1^. Solvents: A, B; compositions: A1A^−1^2 (for details, see the Supporting Information, Figure S1).

*Nano-HPLC*: LC Packings Ultimate Nano-HPLC System with Dionex UVD 340S diode array detector for LC-MS experiments. Injection loop 1 μL (Rheodyne injection valve). Column: Nucleosil 125–5 50 mm×100 μm i.d. capillary column at room temperature. Flow rate 300 nL min^−1^. Solvents: C, D; compositions: N1/N2 (for details, see the Supporting Information, Figure S1).

*HPLC-SPE:* the HPLC system was coupled by a rheodyne injection valve without loop to a Gynkotek M300 Pump with a steady water flow of 2.5 mL min^−1^ connected to a RP-18 cartridge (Thermo ODS 14 mm x 10 mm i.d. pre-column). Data were registered and processed with Chromeleon V6.50.

*Preparative HPLC:* Gynkotek HPLC System with manual sampler, M300 pump, UVD 340 diode array detector and Jasco FP-920 fluorescence detector, a Hypersil ODS 5 μm 250 mm × 21.2 mm i.d. column, at room temperature, flow rate of 5 mL min^−1^. Solvents A, B; composition: isocratic solvent mixtures with 45 to 55 % of MeOH.

*pH values*: Measured by using a WTW Sentix 21 electrode connected to a WTW pH 535 digital pH meter.

**Spectroscopy**

*UV/Vis:* Hitachi U-3000. *CD:* Jasco J715.

*Fluorescence*: Varian Cary Eclipse.

*NMR spectroscopy:* ^1^H- and ^13^C NMR*:* Bruker UltraShield 600 MHz or Varian Unity Inova 500 MHz spectrometers; ^13^C NMR (125 MHz, CD_3_OD, ^13^C-signal assignments from HSQC and HMBC-correlations).[51,52]

*Mass spectrometry:* Finigan MAT 95-S, positive-ion mode, *m*/*z* (rel. abundance); ESI-MS:[48] infusion, spray voltage 1.4 kV, solvent water/MeOH 1:1 (v/v). FAB-MS:[49] cesium gun, 20 keV, glycerol or 3-nitrobenzylic alcohol (NOBA) matrix; High resolution (HR) mass spectra: polyethylene glycol as internal mass standard.

**Isolation of** ***Mc*****-FCCs and** ***Mc*****-NCCs**: Ninety-nine freshly ripe bananas (about 20 kg, *Musa acuminata*, Cavendish cultivar, Chiquita brand) from the supermarket in Innsbruck were worked up as follows: the outer parts of the peels were blended with liquid nitrogen in a beaker. MeOH (1600 mL) and potassium phosphate buffer pH 7 (500 mL of 100 mm) were added. The mixture was filtrated over celite. The solid residue was again blended with MeOH (700 mL) and buffer (200 mL), the filtration was repeated. The filtrates were concentrated to 1 L on a rotary evaporator. The deeply colored solution was again filtrated over celite; the residue was washed with water. The clear solution was applied to an RP18-column (6 g of material in water), washed with water (30 mL) and eluted with MeOH (200 mL). MeOH was evaporated on a rotary evaporator and water (40 mL) was added. The solution was acidified with HCO_2_H (85 %, 150 μL) and extracted three times with dichloromethane (150 mL). The organic phases were dried on a rotary evaporator, the dry residue was dissolved in MeOH (2 mL) and the solution was neutralized with potassium phosphate buffer pH 7 (10 mL of 100 mm). The extract was separated by preparative HPLC. Fractions were collected on ice and in darkness and stored at −80 °C. Fractions of interest were repeatedly purified by semi-preparative HPLC-SPE and were trapped on a RP-18 cartridge, rinsed with 10–15 mL of water and eluted with MeOH/water 9:1 (v/v, 4 mL), solvents were evaporated in vacuo and dried samples were stored sealed under Ar atmosphere in a −80 °C freezer.

A second batch of freshly ripe 65 bananas ("Dole" brand, about 13 kg) was worked up in an analogous way, but by using MeOH/potassium phosphate buffer pH 7, 3:1 (v/v, 1600 mL) for extraction, to obtain a sample of *Mc*-NCC-42 for spectroscopic analysis.

For further mass spectrometric analysis with nano-HPLC-ESI-MS the outer parts of frozen peels of 9 yellow bananas were crushed. MeOH (150 mL) and potassium phosphate buffer pH 7.0 (50 mL of 100 mm) were added, and the mixture was subjected to filtration. The filtrate was evaporated to about 20 % of its volume at ambient temperature and filtrated again. The filtrate was used for characterization by nano-HPLC-ESI-MS of *Mc*-NCC-61, *Mc*-NCC-49 and *Mc*-NCC-42 (solvent composition N2) and *Mc*-NCC-59, (solvent composition N1).

**Spectroanalytical data**

Mc-FCC-62: ^1^H NMR (CD_3_OD, 0 °C, 500 MHz): *δ*=1.12 (d, *J*=7.3 Hz, H_3_C(18^1^)), 1.75 (m, H_A_C(17^1^)), 1.95 (m, H_B_C(17^1^)), 2.05 (s, H_3_C(2^1^)), 2.17 (s, H_3_C(12^1^)), 2.24 (s, H_3_C(7^1^)), 2.34 (m, H_2_C(17^2^)), 2.41 (m, HC(17)), 2.57 (dd, *J*=7.8/18.2 Hz, H_A_C(20)), 2.65 (m, H_2_C(8^1^)), superimposed by 2.66 (m, HC(18)), 3.03 (dd, *J*=4.4/18.2 Hz, H_B_C(20)), 3.50 (m, H_2_C(8^2^)), 3.73 (s, H_3_C(13^5^)), 4.02 (s, H_2_C(10)), 4.63 (m, HC(1)), 5.39 (dd, *J*=2.2/11.7 Hz, H_A_C(3^2^)), 6.21 (d broad, *J*=17.0 Hz, H_B_C(3^2^)), 6.53 (dd, *J*=11.8/17.6 Hz, HC(3^1^)), 9.37 ppm (s, HC(5)). ^13^C NMR (CD_3_OD, 0 °C, 500 MHz):*δ*=9.10 (7^1^), 9.33 (12^1^), 12.7 (2^1^), 18.4 (18^1^), 23.3 (10), 27.8 (8^1^), 47.7 (17), 52.4 (18), 53.0 (13^5^), 55.3 (17^1^), 58.2 (1), 62.5 (8^2^), 113 (12), 119 (3^2^), 122 (8), 123 (3^1^), 127 (13), 130 (3), 130 (6), 135 (7), 137 (11), 138 (9), 157 (2), 171 ppm (19) (see the Supporting Information, Figure S1); UV/Vis: *λ*_max_ (*ε*_rel_; solvents: MeOH/potassium phosphate buffer, pH 7, 55/45 (v/v) 100 mM)=248 (sh, 1.00), 314 (0.81), 362 (0.41); HR-FAB (matrix glycerol): *m*/*z* calcd for C_35_H_41_N_4_O_8_: 645.292 [*M*+H]^+^; found: 645.314. See Figure 7 for the molecular constitution of ***Mc*-FCC-62**.

**7:**
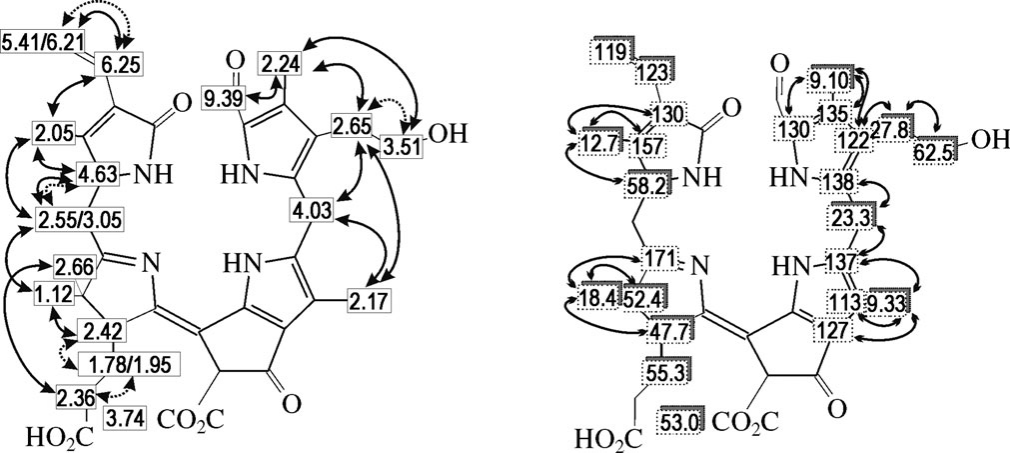
Molecular constitution of *Mc*-FCC-62 derived from 2D NMR data (500 MHz, CD_3_OD, 0 °C). Left: graphical representation of homonuclear ^1^H,^1^H correlations, arrows with dotted lines represent correlations from COSY, bold arrows represent correlations from ROESY; right: hetero-nuclear ^13^C,^1^H correlations, shadowed boxes indicate assignments from direct correlations, obtained from HSQC-spectra, arrows indicate assignments from long range coupling, obtained from HMBC-spectra.

***Mc*****-FCC-71**: UV/Vis: *λ*_max_ (*ε*_rel_, in methanol)=262 (sh, 0.79), 317 (1.00), 359 nm (0.65). For ^1^H- and ^13^C NMR and ESI-MS, see the Supporting Information.

***Mc*****-NCC-61**: *t*_R_=61 min; ^1^H NMR (CD_3_OD, 500 MHz): *δ*=1.93 (s, H_3_C(18^1^)), 1.96 (s, H_3_C(2^1^)), 2.13 (s, H_3_C(12^1^)), 2.25 (s, H_3_C(7^1^)), 2.33 (m, H_2_C(17^2^)), 2.48 (dd, *J*=9/14.5 Hz, H_A_C(20)), 2.62 (m, H_A_C(17^1^) superimposed by H_2_C(8^1^)), 2.71 (m, H_B_C(17^1^)), 2,85 (dd, *J*=5/14.5 Hz, H_B_C(20)), 3.48 (m, H_2_C(8^2^)), 3.75 (s, H_3_C(13^5^)), 3.94 (s, H_2_C(10)) superimposed by 3.95 (m, HC(1)), 5.35 (dd, *J*=2.1/11.5 Hz, H_A_C(3^2^)), 6.09 (dd, *J*=2.1/17.6 Hz, H_B_C(3^2^)), 6.44 (dd, *J*=11.5/17.6 Hz, HC(3^1^)), 9.34 ppm (s, HC(5)); ^13^C NMR (CD_3_OD, 500 MHz): *δ*=111.1 (12), 114.1 (18), 119.3 (17), 119.4 (8), 123.4 (19), 124.8 (13), 127.6 (3), 128.2 (6), 132.8 (11), 155.7 (2), 170.4 ppm (13^3^); UV/Vis: *λ*_max_ (*ε*_rel_, in methanol)=245 (1.00), 313 (0.89); MS (nano-HPLC-ESI, solvent: ACN/water, gradient N_2_): *m*/*z* (rel. abundance)=699.20 (3, [*M*+2Na]^+^), 683.19 (19, [*M*+K]^+^), 667.21 (27 [*M*+Na]^+^), 646.19 (40), 645.21 (100, [*M*+H]^+^), 613.19 (12, [*M*-CH_3_OH+H]^+^), 522.10 (5, [*M*-ring A+H]^+^). HR-FAB (matrix NOBA): *m*/*z* calcd for C_39_H_41_N_4_O_8_: 645.292 [*M*+H]^+^; found: 645.291.

***Mc-*****NCC-59**: (*t*_R_=59 min); UV/Vis (online DAD spectrum with MeOH/100 mM potassium phosphate buffer, pH 7, about 1/1 (v/v)): *λ*_max_ (*ε*_rel_)=243 (1.00), 314 (0.87). MS (nano-HPLC-ESI): *m*/*z* (rel. abundance)=829.33 (14, [*M*+Na]^+^), 809.35 (14), 808.35 (48), 807.35 (100, [*M*+H]^+^), 775.32 (6, [*M*-CH_3_OH+H]^+^), 732.22 (6), 684.22 (8, [*M*-ring A+H]^+^), 661.21 (6), 645.22 (22, [*M*-C_6_H_10_O_5_+H]^+^), 613.17 (5, [*M*-C_6_H_10_O_5_-CH_3_OH+H]^+^); HR-FAB matrix: glycerol: *m*/*z* calcd for C_41_H_51_N_4_O_13_^+^: 807.345 [*M*+H]^+^; found: 807.342.

***Mc*****-NCC-58**: (*t*_R_=58 min); UV/Vis: *λ*_max_ (*ε*_rel_, in MeOH)=242 (1.00), 314 (0.82). CD (*c*=2×10^−5^ M, in MeOH): *λ*_max/nm_(Δ*ε*)=222 (21.2), 245 (−5.4), 283 (−12.0), 316 (6.3); ESI-MS: *m*/*z* (%): 945.23 (6, [*M*+3 K]^+^), 929.30 (7, [*M*+2 K+Na]^+^), 907.25 (18, [*M*+2 K]^+^), 891.32 (24, [*M*+K+Na]^+^), 871.31 (29), 870.28 (57), 869.31 (100, [*M*+K]^+^), 854.32 (23), 853.37 (39, [*M*+Na]^+^), 833.28 (14), 832.30 (42), 831.37 (63, [*M*+H]^+^).

***Mc*****-NCC-55**: (*t*_R_=55 min); UV/Vis: *λ*_max_(*ε*_rel_, in MeOH)=242 (1.00), 314 (0.84). CD (*c*=1.06×10^−5^ M, in MeOH): *λ*_max/nm_ (Δ*ε*)=230 (−16.1), 283 (6.9), 320 (−5.0); MS (ESI): *m*/*z* (%)=891.32 (9, [*M*+K+Na]+), 869.31 (30, [*M*+K]^+^), 853.31 (30 [*M*+Na]^+^), 833.39 (19), 832.35 (55), 831.31 (100,[*M*+H]^+^), 705.25 (13), 683.25 (13), 667.21 (6, [*M*-C_7_H_7_O_6_ +Na]^+^).

***Mc*****-NCC-49**: (*t*_R_=49 min): ^1^H NMR (CD_3_OD, 0 °C, 500 MHz): *δ*=1.92 (s, H_3_C(18^1^)), 1.94 (s, H_3_C(2^1^)), 2.10 (s, H_3_C(12^1^)), 2.24 (s, H_3_C(7^1^)), 2.37 (m, H_2_C(17^2^)), 2.45 (dd, *J*=10.0/14.0 Hz, H_A_C(20)), 2.63 (m, H_2_C(8^1^)), superimposed by 2.66 (m, H_A_C(17^1^)), 2.78 (m, H_B_C(17^1^)), 2,83 (dd, *J*=4.1/14.0 Hz, H_B_C(20)), 3.46 (m, H_2_C(8^2^)), 3.92 (s, H_2_C(10)), 3.98 (m, HC(1)), 5.34 (d broad, *J*=11.5 Hz, H_A_C(3^2^)), 6.08 (d broad, *J*=17.0 Hz, H_B_C(3^2^)), 6.42 (dd, *J*=11.5/17.0 Hz, HC(3^1^)), 9.34 ppm (s, HC(5)); UV/Vis: *λ*_max_ (*ε*_rel_, in MeOH)=242 (sh, 1.00), 313 nm (0.90); MS (nano-HPLC-ESI): *m*/*z* (rel. abundance)=677.23 (2), 659.18 83), 633.18 (6), 632.18 (17), 631.17 (100, [*M*+H]^+^), 613.15 (7, [*M*-H_2_O+H]^+^), 609.16 (6), 588.17 (20), 587.16 (39, [*M*-CO_2_+H]^+^), 508.06 (4, [*M*-ring A+H]^+^), 464.11 (3), 424.96 (2); HR-FAB matrix: glycerol: *m*/*z* calcd for C_34_H_39_N_4_O_8_^+^: 631.276 [*M*+H]^+^; found: 631.279.

***Mc*****-NCC-42**: (*t*_R_=42 min): UV/Vis (online DAD spectrum in MeOH/100 mM potassium phosphate buffer, pH7, about 2/3 (v/v)): ^1^H NMR (CD_3_OD, 0 °C, 500 MHz): *δ*=1.93 (s, H_3_C(18^1^)), 2.02 (s, H_3_C(2^1^)), 2.08 (s, H_3_C(12^1^)), 2.26 (s, H_3_C(7^1^)), 2.32 (t, *J*=7 Hz, H_2_C(17^2^)), 2.54 (dd, *J*=8.5/14.6 Hz, H_A_C(20)), 2.62 (m, H_A_C(17^1^)) superimposed by 2.63 (dd, H_2_C(8^1^)), 2.72 (m, H_B_C(17^1^)), 2,87 (dd, *J*=4.4/14.6 Hz, H_B_C(20)), 3.47 (m, H_2_C(8^2^)), 3.62 (dd, *J*=5.0/11.3 Hz, H_A_C(3^2^)), 3.69 (dd, *J*=6.6/11.3 Hz, H_B_C(3^2^)), 3.75 (s, ester methyl), 3.97 (d, *J*=3 Hz, H_2_C(10)), 4.01 (dd, *J*=4.5/8.6 Hz, HC(1)), 4.56 (dd, HC(3^1^)), 9.36 ppm (s, HC(5)). ^13^C NMR (CD_3_OD, 0 °C, 500 MHz): *δ*=8.93 (7^1^), 9.16 (12^1^), 9.31 (18^1^), 12.4 (2^1^), 21.9 (17^1^), 23.8 (10), 28.1 (8^1^), 29.7 (20), 37.2 (15), 39.1 (17^1^), 52.8 (13^5^), 62.4 (1), 62.5 (8^2^), 65.9 (3^2^), 68.6 (3^1^), 113 (12), 115 (18), 120 (17), 121 (8), 124 (16), 124 (19), 126 (13), 129 (4), 132 (3), 135 (7), 135 (11), 139 (9), 159 (2) ppm (see Figure 8); *λ*_max_ (ε_rel_)=248 (0.77), 319 nm (1.00); Nano-HPLC-ESI-MS: *m*/*z* (rel. abundance)=718.19 (8), 717.23 (17, [*M*+K]^+^), 702.21 (15), 701.22 (39 [*M*+Na]^+^), 681.26 (11), 680.25 (40), 679.24 (100, [*M*+H]^+^), 648.21 (18), 647.18 (12, [*M*-CH_3_OH+H]^+^), 522.12 (24, [*M*-ring A+H]^+^); HR-FAB matrix: glycerol: *m*/*z* calcd for C_35_H_43_N_4_O_10_^+^: 679.297 [*M*+H]^+^; found: 679.300.

**8:**
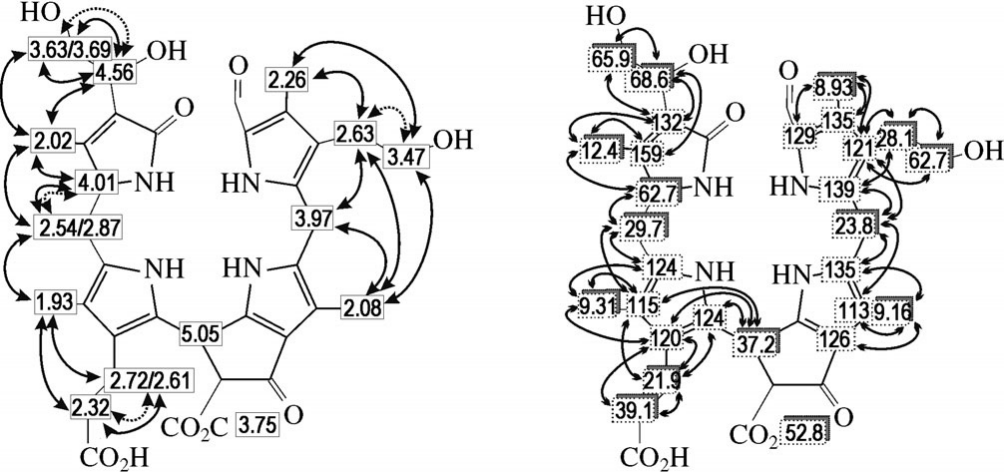
Molecular constitution of *Mc*-NCC-42 derived from 2D NMR data (500 MHz, CD_3_OD). Left: graphical representation of homonuclear ^1^H,^1^H-correlations, arrows with dotted lines represent correlations from COSY, bold arrows represent correlations from ROESY; right: heteronuclear ^13^C,^1^H correlations, shadowed boxes indicate assignments from direct correlations, obtained from HSQC-spectra, arrows indicate assignments from long-range coupling, obtained from HMBC-spectra.

***Mc*****-NCC-26**: (*t*_R_=26 min): UV/Vis (DAD spectrum in MeOH/100 mM potassium phosphate buffer, pH7, ≍3/7 (v/v)): *λ*_max_ (ε_rel_)=246 (sh, 0.81), 316 nm (1.00); FAB-MS: *m*/*z* (rel. abundance)=666.14 (40), 665.10 (100, [*M*+H]^+^).

**Citric acid induced isomerization of** ***Mc*****-FCC-56**: Aqueous citric acid (1 M, 60 μL) was added to a solution of *Mc*-FCC-56 (3 mL of a 30.9 μm solution) and *Mc*-FCC-53 (3:1 mixture of FCC-isomers) in MeOH. The mixture was stored for 159 h under an Ar atmosphere in the dark at 15 °C. Water (1 mL) then was added and the resulting solution was subjected to analytical HPLC. Complete disappearance of FCCs and appearance of two major NCCs were indicated (see the Supporting Information, Figure S2). The two most abundant NCCs (*Mc*-NCC-55 and *Mc*-NCC-58) were isolated by semi-preparative HPLC and their mass spectra, CD spectra (see Figure 4) and UV/Vis-spectra (see the Supporting Information, Figure S3) were recorded. They were identified by HPLC-analysis (co-elution experiments) with authentic *Mc-*NCC-55 and *Mc-*NCC-58 (see the Supporting Information, Figure S4 and S5).

**Co-elution experiments** ***Mc*****-NCC-42 with** ***Hv***-**NCC-1 and** ***So***-**NCC-2**: The most abundant NCC fraction found in extracts of yellow-banana peels, *Mc*-NCC-42, was isolated and characterized (as described above). The purified *Mc*-NCC-42 sample was applied to analytical HPLC, as were 1:1 mixtures of *Mc*-NCC-42 with *Hv*-NCC-1[8] and *So*-NCC-2,[32] respectively. These standardized analytical HPLC-experiments identified *Mc*-NCC-42 (*t*_R_=42) with *So*-NCC-2 (*t*_R_=42), whereas it showed *Mc*-NCC-42 to differ from *Hv*-NCC-1 (*t*_R_=40) (*t*_R_=retention time (in min); see the Supporting Information, Figure S6).

**Analysis of FCCs in ripening bananas during storage**: Ethylene-ripened green–yellow bananas (at rs=5) were stored at ambient temperature and a normal daylight night rhythm. Banana peels were extracted at day zero, one, two, three and four. The extracts were produced from 6–8 cm^2^ of the peel, ground with liquid nitrogen in a mortar. MeOH (500 μL) and potassium phosphate buffer (200 μL of 100 mm, pH 7) were added in the mortar. The mixture was then ultra-filtrated (5 min, 13 000 rpm, regenerated cellulose); 150 μL of the filtrate were applied to the analytical HPLC system. Relative amounts of FCCs were determined by integration of the peaks of the luminescence trace resulting from an FCC; the area was normalized according to the area of peel used for extraction (and was averaged over three to five experiments, see Figure 5).
